# Fabrication of TiO_2_-Embedded Polyimide Layer with High Transmittance and Improved Reliability for Liquid Crystal Displays

**DOI:** 10.3390/polym13030376

**Published:** 2021-01-26

**Authors:** Seung-Rak Son, Jongil An, Jin-Wook Choi, Jun Hyup Lee

**Affiliations:** Department of Chemical Engineering, Soongsil University, Seoul 06978, Korea; tmdfkr0128@naver.com (S.-R.S.); godzzi154@nate.com (J.A.); cjw10546@naver.com (J.-W.C.)

**Keywords:** hybrid polyimide, liquid crystal display, reliability, TiO_2_ nanoparticle, transmittance

## Abstract

Construction of liquid crystal (LC) alignment by introducing polyimide (PI) to indium tin oxide (ITO) electrodes is one of the main methods to realize high-resolution images in liquid crystal displays (LCD). However, the loss of transmittance caused by the difference in refractive index between ITO and PI leads to direct degradation of LCD performance. Thus, we herein fabricated a functional hybrid PI alignment layer that reduces the difference in refractive index and greatly increases the transmittance of the device by introducing inorganic titanium dioxide (TiO_2_) nanoparticles (NP) to the organic PI. The highly refractive TiO_2_ NPs were surface-treated with stearic acid comprising long alkyl chains to improve their dispersibility and uniformly dispersed in the PI matrix by simply stirring the mixture. The hybrid PI mixture was spin-coated on the ITO substrate, and the resulting LC cell exhibited excellent electro-optical properties. In addition, the reliability of the LC cells was enhanced by the inclusion of the TiO_2_ NPs, which was confirmed through the evaluation of voltage holding ratio, residual direct current, and LC cell reliability. Overall, functional hybrid PI can be used in advanced display technology for next-generation LC devices that require high transmittance and reliability.

## 1. Introduction

Currently, the liquid crystal display (LCD) is widely used in various applications such as smartphones, monitors, cameras, and other devices [1,2,3]. In order to drive the device, the liquid crystal (LC) molecules must be controlled by the applied voltage [4,5]. In addition, a vertically aligned LC device with good display performance can only be implemented when the initial orientation of the LCs is uniformly aligned. One of the most important electro-optical properties of a LC-based display device is transmittance. For this reason, indium tin oxide (ITO), a transparent electrode, is used as the electrode of the display. The ITO electrode is used not only in LCDs but also in many displays such as organic light emitting diodes (OLED) and quantum dot light emitting diodes (QLED) [6,7,8,9]. In addition, the alignment layer that determines the initial orientation of the LC is also important, and the most commonly used material for this layer is polyimide (PI) [10,11,12,13,14,15,16]. The LC can be uniformly aligned along a desired direction through the interactions of the LC molecules at the molecular level. However, the commonly used PI lowers the optical transmittance of the device due to a mismatch in its refractive index with that of the ITO substrate. In addition to the problem of the lowered transmittance, the electro-optical properties of the device may be lowered due to the free ions generated by impurities during the synthesis of the LC [17,18]. These free ions move between the electrodes and induce a screening effect, thereby degrading the performance of the device [19].

**Figure 1:**
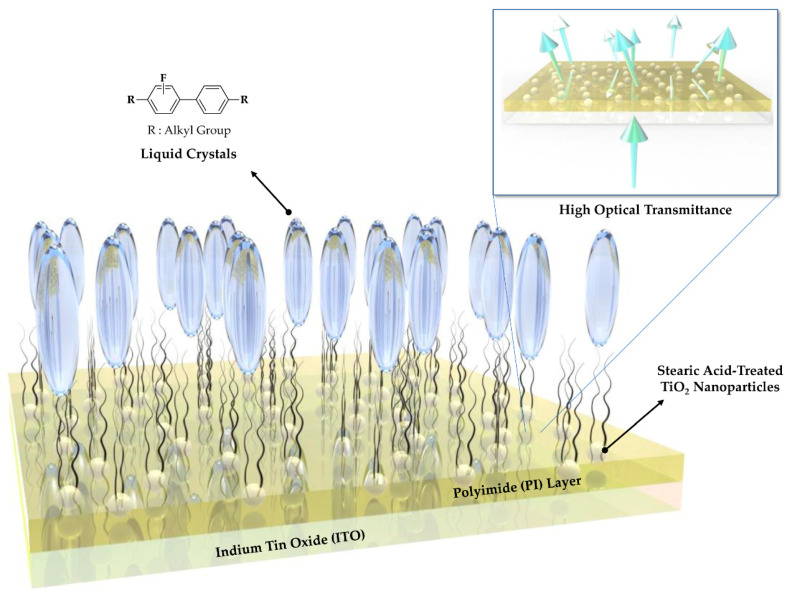
Schematic diagram of TiO_2_-embedded hybrid polyimide layer for liquid crystal displays.

Therefore, we herein propose a facile and effective method to capture the free ions in LCs to suppress the screening effect while simultaneously increasing the transmittance of the device. Introducing stearic acid-treated TiO_2_ nanoparticles (NPs) into the PI polymer can solve the problems presented above (Figure 1). The long hydrophobic alkyl chains in the stearic acid improve the vertical alignment capability of the LC, which in turn improves the controllability of the LC by the applied voltage. In addition, TiO_2_ has a high refractive index of 2.6, and thus the optical transmittance of the device can be increased by the closely matched refractive index of the PI layer (1.6) with that of the ITO substrate (1.8). Moreover, the screening effect can be prevented because of the ability of the TiO_2_ NPs to trap the free ions of the LC [20,21,22]. The effects of the TiO_2_ NPs introduced into PI were evaluated by using two sizes of NPs (15 and 300 nm) and different NP contents. The functional hybrid PI layer was found to improve not only the optical performance but also the reliability of the manufactured LC device. As mentioned earlier, LC-based devices used in televisions and monitors must achieve the same performance even under long-term operation. A problem of existing LCDs is the phenomenon of an image sticking from long-term operation [23,24,25,26,27]. In addition to suppressing the screening effect and preventing the generation of a residual direct current (DC) voltage, the introduced TiO_2_ NPs also maintained the vertical alignment ability of hybrid PI layer due to the effects of the long hydrophobic alkyl chains of stearic acid. Improved electro-optical properties of the device can be obtained by a simple mixing process, and thus the proposed method can be implemented easily to produce a competitive alignment layer in displays requiring high transmittance and reliability.

## 2. Materials and Methods

### 2.1. Preparation of Functional Hybrid Polyimide Layer

Various levels of surface-treated inorganic TiO_2_ NPs (Cosmax, Seongnam, Korea) were added to the organic PI in *N*-methyl-2-pyrrolidone (AL607XX, JSR, Tokyo, Japan) and then stirred for 24 h at 25 °C. The sizes of the surface-treated TiO_2_ NPs were 15 and 300 nm and the mass ratios of NPs to PI solution were 0.01, 0.05, 0.1, and 0.5 wt%. The NPs were well dispersed in the PI solution due to the organic alkyl chains of stearic acid. The prepared PI mixtures of each ratio were coated on the ITO substrates and then thermally cured at 200 °C for 1 h. Thus, the functional hybrid PI alignment layer with a thickness of approximately 100 nm was prepared [28,29].

### 2.2. Fabrication of the LC Cell Containing the Functional Hybrid Polyimide Alignment Layer

Prior to introducing the ITO substrate (0.02 kΩ/cm^2^, 0.7 mm, Fine Chemical Industry, Seoul, Korea) as an electrode of the LC cell, the following cleaning process was performed. The substrate was sequentially washed with acetone (97%, Junsei Chemicals, Tokyo, Japan), isopropyl alcohol (98%, Daejung Chemicals, Siheung, Korea), and de-ionized (DI) water for 10 min under ultrasonication and then dried for 15 min on a hot plate at 100 °C. The cleaned substrates were used as the upper and lower electrodes of the cell. Then, the mixtures containing 0.01, 0.05, or 0.1 wt% of TiO_2_ NPs were uniformly spin-coated onto the ITO electrodes to act as the LC alignment layer. The homogeneous hybrid PI layer was prebaked at 80 °C and then imidized at 200 °C for 1 h. To maintain the LC cell gap at 5.25 μm, a spacer (SP-205XX, Sekisui Chemical, Tokyo, Japan) was used, and the upper and lower substrates were sealed using UV curable sealant (SP-25XX, Sekisui Chemical, Tokyo, Japan). The applied sealant was cured by UV irradiation at an energy of 3 J/cm^2^. After the sealant curing process, the nematic LCs comprising fluorobiphenyl derivatives (*T*_NI_ = 75 °C, ∆*n* = 0.095, ∆*ε* = −3.1, Merck, Darmstadt, Germany) was injected into the LC cell by capillary force and annealed at 100 °C for 1 h.

### 2.3. Characterization

**Figure 2:**
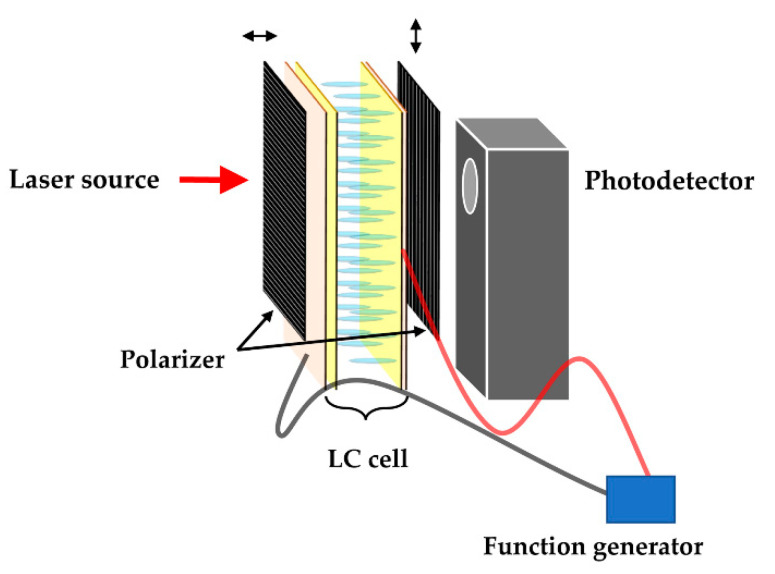
Schematic diagram of electro-optical measurement system.

Polarized optical microscopy (POM; BX51, Olympus, Tokyo, Japan) was performed to investigate the alignment properties of the LCs. Contact angle measurements (Phoenix MT-A, SEO, Suwon, Korea) were carried out to examine the effect of the surface-treated TiO_2_ NPs on the hydrophobic properties of hybrid PI layer. Atomic force microscopy (AFM) was conducted in contact mode with silicon tips using a PSIA XE-100 (Park systems, Suwon, Korea). To examine the optical transmittance of the LC cell, ultraviolet-visible spectroscopy (UV–vis; Mega-900, Scinco, Seoul, Korea) was performed. To confirm the electro-optical performance of the manufactured LC cells, the voltage-transmittance (V-T) curves and response times were recorded using an electro-optical measurement system comprising a laser light source (1135P, JDSU, Milpitas, CA, USA), a photodetector (ET-2000, EOT, Edinburgh, UK), a function generator (33210A, Agilent, Santa Clara, CA, USA), and an oscilloscope (TBS1062, Tektronix, Beaverton, OR, USA). The test LC cell was aligned between the laser light source and the photodetector with crossed polarizers, and then the electro-optical measurement was carried out using a function generator (LC cell) and an oscilloscope (photodetector) (Figure 2). The voltage holding ratio (VHR) and residual direct current (RDC) of the LC cell were evaluated using an LC characteristic measurement system (Model 6254, Toyo, Tokyo, Japan).

## 3. Results and Discussion

**Figure 3:**
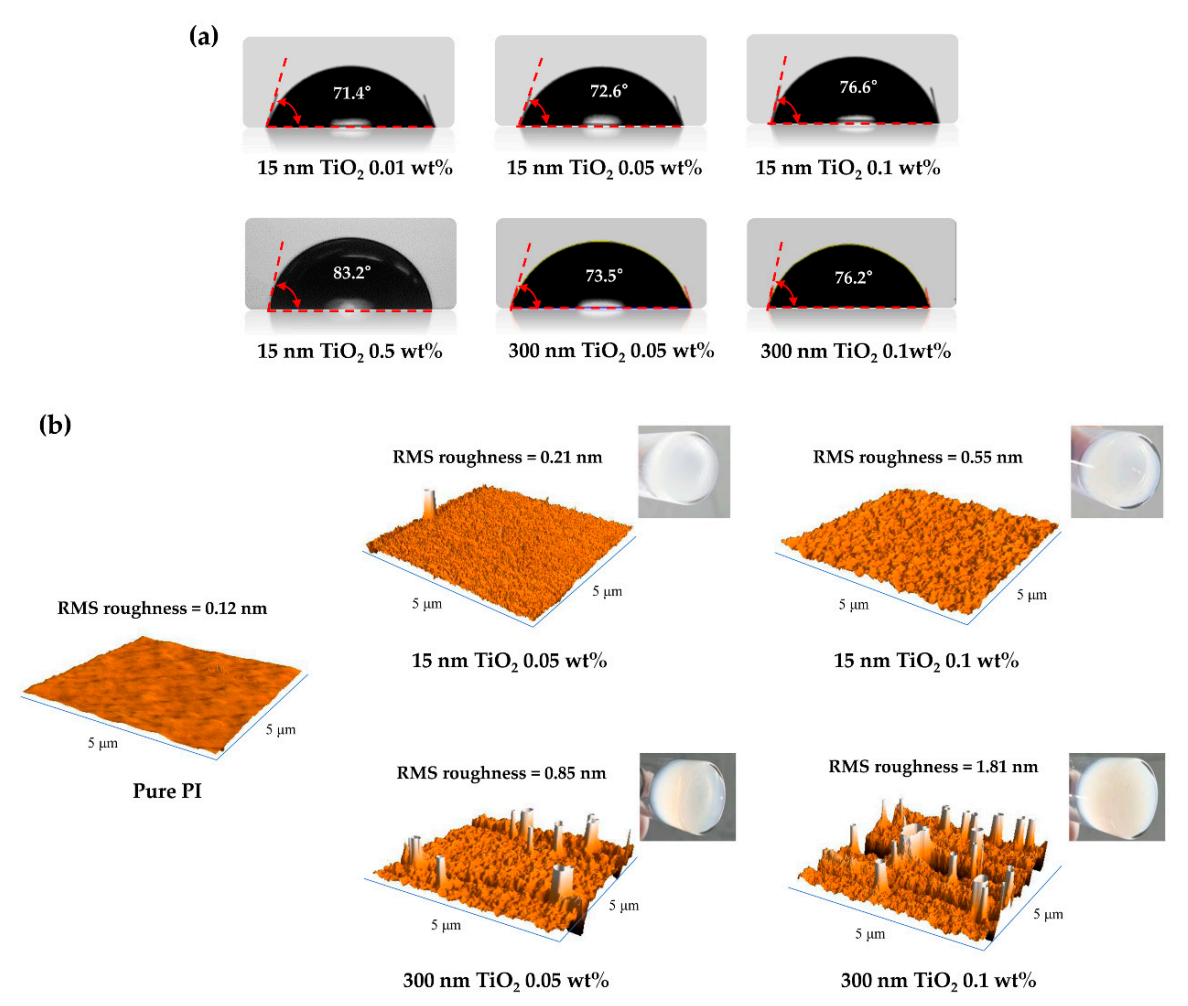
(**a**) Photographic images of the contact angles for distilled water on the indium tin oxide (ITO) substrates with hybrid polyimide layers. (**b**) Atomic force microscopy (AFM) three-dimensional view and photographic images of the pure polyimide and hybrid polyimide layers according to TiO_2_ content and size.

To determine the effect of the surface-treated TiO_2_ nanoparticles on the hydrophobicity of functional hybrid PI layer, contact angle measurements were performed. Figure 3a shows the water contact angles for each content of TiO_2_ NPs of 15 and 300 nm in size. The contact angles of the functional hybrid PI layer were 71.4, 72.6, 76.6, and 83.2° at 0.01, 0.05, 0.1, and 0.5 wt%, respectively, for 15-nm-TiO_2_. Additionally, the contact angles of the hybrid PI layer containing 0.05 and 0.1 wt% 300-nm-TiO_2_ were 73.5 and 76.2°, respectively. Notably, the water contact angles of the hybrid PI layers were higher than that of pure PI layer (70°). From these results, it was confirmed that the water contact angle of the hybrid PI layer increased as the TiO_2_ NP content increased. These results are attributed to the influence of the long hydrophobic alkyl chains of stearic acid on the TiO_2_ surface. As the content of surface-pretreated TiO_2_ increases, the hybrid PI layer coated on the ITO substrate becomes more hydrophobic and the contact angle of DI water, which is the test sample, increases. As a result, it was confirmed that the surface of TiO_2_ was treated with stearic acid, and the change in the surface energy of each substrate was confirmed. In addition, Figure 3a shows the trend of the contact angle due to the two sizes of TiO_2_ NPs, where the influence of stearic acid was once again confirmed. As mentioned above, as the TiO_2_ content increased, the contact angle also increased, and this trend was the same for both TiO_2_ dimensions (15 and 300 nm). Furthermore, when the TiO_2_ content was the same, the contact angles were similar even if the size of the TiO_2_ NPs was different, confirming that the alkyl chains of stearic acid, not the size of the TiO_2_ particles, dominantly affected the change in the surface energy. Importantly, the long alkyl chains of stearic acid attached to the NP surface interacted with the LC molecules to induce vertical alignment of the LC molecules.

AFM and solubility measurements were performed to confirm the dispersibility of the TiO_2_ NPs in the PI layer. From the photographic images of the hybrid PI solutions, it is confirmed that the TiO_2_ NPs are well dispersed in the PI solution without precipitation of particles, as shown in Figure 3b. Moreover, the three-dimensional view images of AFM show that 15-nm-TiO_2_, which is smaller than PI layer, has a uniform surface morphology without particle aggregations, and the surface roughness increases as the TiO_2_ content increases, indicating the uniform dispersion of the TiO_2_ in the PI layer. In case of 300-nm-TiO_2_, since the size of TiO_2_ is larger than thickness of PI layer (100 nm), the protrusions of TiO_2_ NPs were found, but their sizes were similar to that of TiO_2_ particle. Furthermore, as the content increased, the number of protrusions increased in a uniform manner. These results suggest that the surface-pretreated TiO_2_ NPs can afford high dispersibility in the PI layer.

**Figure 4:**
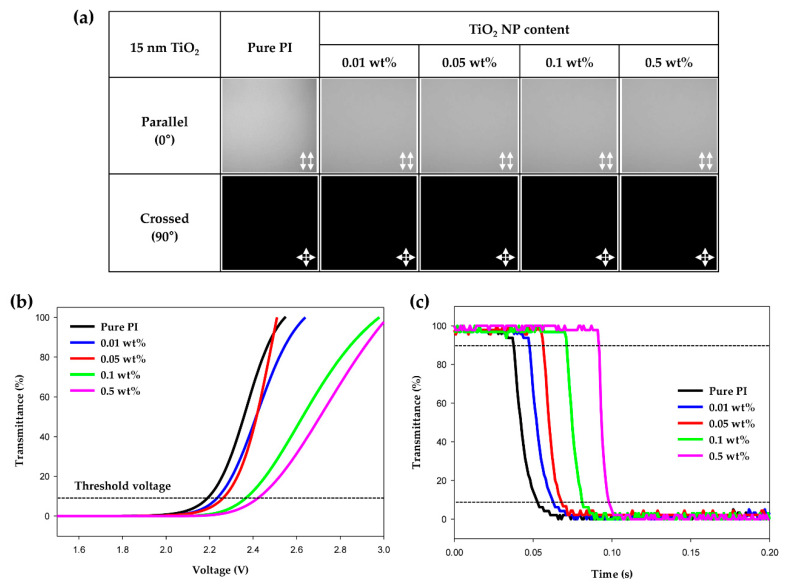
(**a**) Polarized optical microscopy (POM) images of the fabricated liquid crystal (LC) cells with pure polyimide and hybrid polyimide layers containing 15-nm-TiO_2_. (**b**) Voltage-transmittance (V-T) curves and (**c**) response time curves of the LC cells with hybrid polyimides and pure polyimide.

POM images of the LC cells with pure PI and hybrid PI alignment layers were obtained to confirm the vertical alignment ability of the LCs due to the introduction of the improved alignment layer. Pure PI, which is conventionally used as the LCD alignment layer, shows a clear image with parallel and cross polarizers. In other words, the pure PI alignment layer vertically aligned the initial direction of the LCs. As shown in Figure 4a, the LC cells containing the hybrid PI layer (prepared using the 15-nm-TiO_2_ NPs) also provided clear images. The clarity of the hybrid PI-LC cells under polarization is comparable to that of pure PI-LC cell. From this result, it was confirmed that the new hybrid PI alignment layer does not adversely affect the vertical alignment of the LCs. In addition, as mentioned above, the long alkyl chains of stearic acid on the surface of the TiO_2_ NPs facilitate vertical alignment through their interactions with the LCs. Figure 4b shows the voltage-transmittance (V-T) curves measured to confirm the electro-optical characteristics of the manufactured LC cells. To compare with pure PI, hybrid PI-LC cells were also tested under the same conditions. The threshold voltage value of the pure PI-LC cell was 2.22 V, and those of the hybrid PI-LC cells were 2.24, 2.27, 2.38, and 2.42 V for TiO_2_ contents of 0.01, 0.05, 0.1, and 0.5 wt%, respectively. Here, the threshold voltage is defined as the voltage at 10% transmittance of the LC cell [30,31]. Evidently, the threshold voltage values of the hybrid PI-LC cells were higher than that of the pure PI-LC cell. The reason for the increase in the threshold voltage from that of the pure PI-LC cell is that the alkyl chains of stearic acid contained in the hybrid PI layer interact with the LCs to further increase the vertical alignment of the LCs. The decay time of each cell was measured to determine the response speed, as shown in Figure 4c. The gray-to-gray method, which is most commonly used, was used to measure the response speed. Here, the gray range was designated as dark gray and light gray for a transmittance of 10 and 90%, respectively [32,33,34]. Therefore, the decay time is the time taken to drop from 90 to 10% of the transmittance. The decay time of the pure PI-LC cell was 15 ms, and those of the hybrid PI-LC cells were 15, 11, 10, and 7 ms for TiO_2_ contents of 0.01, 0.05, 0.1, and 0.5 wt%, respectively. It can be seen that the decay time in the hybrid PI-LC cells were faster than that of the pure PI-LC cell, and the higher the TiO_2_ content, the faster the decay time. As a result, it was once again confirmed that the vertical alignment ability of the LC was improved by hybrid PI layer with the surface-treated TiO_2_ NPs.

**Figure 5:**
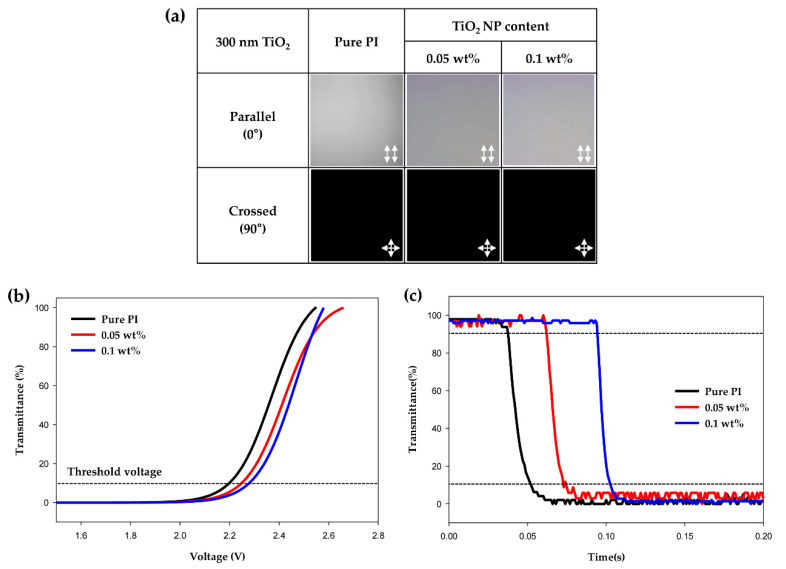
(**a**) Polarized optical microscopy (POM) images of the fabricated cells with pure polyimide and hybrid polyimide layers containing TiO_2_ of 300 nm in size. (**b**) Voltage-transmittance curves and (**c**) response time curves of the hybrid polyimides and pure polyimide LC cells.

To determine the electro-optical characteristics of the cells according to the size of the TiO_2_ NPs, measurements were performed using the same abovementioned method. The size of the TiO_2_ particles increased from 15 nm to 300 nm, and the surface of the TiO_2_ NPs was treated with stearic acid in the same way as the 15-nm-TiO_2_. In addition, two hybrid PIs with TiO_2_ contents of 0.05 and 0.1 wt% were prepared. As shown in Figure 5a, the POM image of the hybrid PI-LC cell with the 300-nm-TiO_2_ is comparable to that of the pure PI-LC cell; clear images were obtained under parallel and crossed polarizations without light leakage. Similar to the results for 15-nm-TiO_2_, 300-nm-TiO_2_ induced a stable vertical alignment of the LCs, and both cells with different TiO_2_ contents showed similar performances. Figure 5b shows the V-T curves of a hybrid PI-LC cell containing 300-nm-TiO_2_. The threshold voltage values of 2.25 and 2.28 V were obtained at TiO_2_ contents of 0.05 and 0.1 wt%, respectively; these values were similar to that of the pure PI-LC cell, but they were slightly lower than those of the hybrid PI-LC cells containing 15-nm-TiO_2_. Specifically, when comparing the threshold voltage values of the two types of cells containing 0.1 wt% of 15-nm-TiO_2_ and 300-nm-TiO_2_, the hybrid PI-LC cell containing 15-nm-TiO_2_ showed a higher value (+ 0.1 V). Although this difference is insignificant, it can be seen as an effect of the particle size because different voltages were obtained at the same TiO_2_ content. Because the size of the particles increases by 20 times (from 15-nm-TiO_2_ to 300-nm-TiO_2_), the amount of 300-nm-TiO_2_ doped in PI is lower compared with the 15-nm-TiO_2_ doped PI, leading to the decrease in vertical alignment ability of the hybrid PI layer. This particle size effect can be seen in detail in the response speed results shown in Figure 5c. The response speed of the prepared cells was 12 and 19 ms at 0.05 and 0.1 wt%, respectively. The decay time of the cell with 0.1 wt% TiO_2_ NPs was approximately two times higher than that of 15-nm-TiO_2_ at 0.1 wt%. This difference, as mentioned above, can be considered due to the particle size effect in which the applied amount of TiO_2_ particles decreases as the size increases. As a result, the particle size effect (15 and 300 nm) was confirmed, and the electro-optical properties of the LC cells containing 300-nm-TiO_2_ were also confirmed to be similar to that of the pure PI-LC cell.

**Figure 6:**
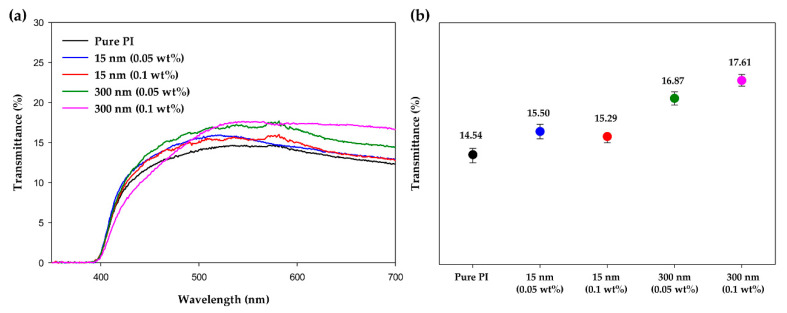
(**a**) UV–vis spectra and (**b**) optical transmittances of the pure polyimide LC cell and LC cells fabricated using hybrid polyimide alignment layer.

To confirm the improved optical transmittance due to TiO_2_ doping, UV–vis spectra were acquired. As can be seen in Figure 6, the increase in transmittance from an LC cell composed of pure PI to the LC cells composed of hybrid PI layers containing TiO_2_ was confirmed. A saturation voltage was applied to the prepared cells to create a white state, and after attaching a polarizing film to the upper and lower substrates of the cells, the transmittance was measured based on air. The transmittance of the pure PI-LC cell at a wavelength of 550 nm was 14.54%. In the same wavelength band, the transmittance values of the hybrid PI-LC cells containing TiO_2_ contents of 0.05 and 0.1 wt% were, respectively, 15.50 and 15.29 for 15-nm-TiO_2_ and 16.87 and 17.61 for 300-nm-TiO_2_. The transmittance of the LC cells containing the hybrid PI alignment layer was higher than that of the pure PI-LC cell. In particular, in the case of the hybrid PI-LC cell containing 0.1 wt% of 300-nm-TiO_2_, an increase from the reference value of the pure PI-LC cell by 20% was observed. The reason for this result is that the hybrid PI alignment layer performs better than the pure PI layer in LC control according to the applied voltage. The long hydrophobic alkyl chains of stearic acid treated on the TiO_2_ NP surface interact with the LCs, and thus LC control is easier. As mentioned earlier, the hybrid PI can suppress the screening effect due to the ion trapping effect of TiO_2_. On the other hand, pure PI without TiO_2_ has a lower optical transmittance than the hybrid PI because the LC receives a relatively lower voltage in the cell than the applied voltage due to the screening effect. In addition, the high refractive index of TiO_2_ can be suggested as an additional reason for the increase in the optical transmittance of the LC cell. Since the conventional pure PI layer has a lower refractive index than the ITO substrate, a loss in light transmittance can be generated due to the total reflection, but the refractive index between the two layers is closely matched due to doping with TiO_2_, which has a high refractive index, thereby increasing the optical transmittance. For these reasons, it was confirmed that the optical transmittance of LC cell was improved by introducing TiO_2_ NPs into PI layer, and the transmittance increased as the size of the TiO_2_ NPs increased.

**Figure 7:**
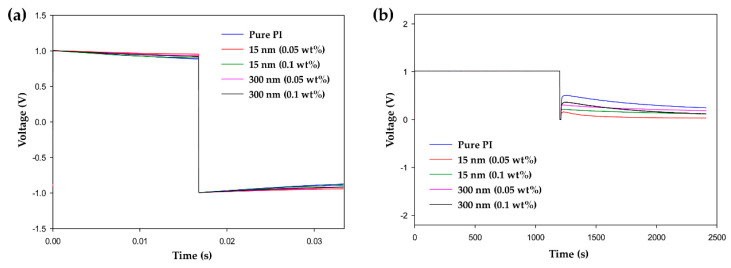
(**a**) Voltage holding ratio (VHR) curves and (**b**) residual direct current (RDC) curves of the LC cells fabricated using pure polyimide and hybrid polyimide alignment layers.

The measurements for voltage holding ratio (VHR) and residual direct current (RDC) were carried out to confirm the reliability performance of the LC cells for long-term operation. The VHR is a function of an LCD device that maintains the applied voltage. A low VHR leads to device performance drawbacks, such as image sticking. Figure 7a shows the VHR curves of the LC cells fabricated with pure PI and hybrid PI layers. The VHR values of the cells containing TiO_2_ contents of 0.05 and 0.1 wt% were, respectively, 97.04 and 94.06% for 15-nm-TiO_2_, and 96.01 and 95.32% for 300-nm-TiO_2_. Compared with the VHR value of the pure PI-LC cell (93.65%), all the hybrid PI-LC cells exhibited higher VHR values. It was confirmed that the VHR value increased by doping PI with TiO_2_ NPs, which can improve the potential image sticking problem that may occur in the display devices. Figure 7b shows the RDC curves of the LC cells containing the conventional PI and the hybrid PI alignment layers. After 2000 s, the RDC value of the pure PI-LC cell was 297.3 mV and those of the hybrid PI-LC cells containing TiO_2_ contents of 0.05 and 0.1 wt% were, respectively, 39.7 and 141.1 for 15-nm-TiO_2_, and 210.9 and 164 mV for 300-nm-TiO_2_. The LC cell containing the hybrid PI alignment layer as a whole showed a lower RDC value than the LC cell with the conventional alignment layer. This low RDC value is a result of the ion trapping effect of TiO_2_ NPs embedded in the PI layer. The LC cell with pure PI as an alignment layer exhibited a relatively high RDC value, which may cause image sticking of the device due to the generation of residual DC voltage. However, the hybrid PI-LC cells significantly lowered the probability of such a disadvantage. Through VHR and RDC measurements, it was confirmed that the introduction of a hybrid PI alignment layer improved the device characteristics for long-term reliability.

**Figure 8:**
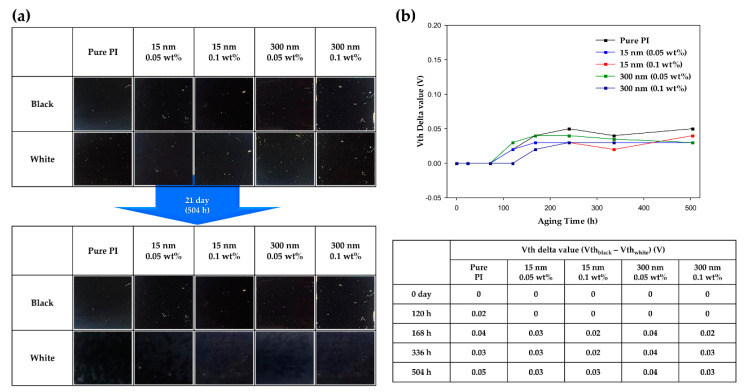
(**a**) Photographic images to evaluate the long-term stability of the LC cells fabricated using pure polyimide and hybrid polyimide over 504 h. (**b**) Difference between the threshold voltages in the white and black states over time.

To evaluate the long-term reliability of the LC cells based on the above RDC and VHR values, the difference between the threshold voltages of the LC cells in the black and white states was measured. Reliability evaluation was conducted over a period of 504 h, and the difference in threshold voltage was measured (Figure 8). It can be seen that the performance of the LCD deteriorated as the difference in threshold voltage increased over time. In the LC cell with pure PI alignment layer, it was confirmed that the delta value of the threshold voltage increased with time. This is because the LC to which the voltage was applied for a long period of time was unstably oriented, and thus the vertical alignment ability was degraded. Therefore, since the voltage was lower than the initial threshold voltage, the threshold voltage delta value increased. By contrast, the LC cell incorporating the hybrid PI as the alignment layer exhibited a lower rate of increase in threshold voltage delta value than the pure PI-LC cell. These results show the same trend as the values of RDC and VHR. Thus, hybrid PI was able to maintain the vertical alignment ability of the LCs, which can be deteriorated by a long-term applied voltage, and it was confirmed that the long alkyl chains of the TiO_2_ NPs formed strong interactions with the LCs. In addition, the screening effect that may occur due to the free ions in the LC was also prevented by the ion trapping effect of TiO_2_, thereby improving the long-term electro-optical performance of the device.

## 4. Conclusions

The introduction of a hybrid PI alignment layer containing inorganic TiO_2_ NPs was successful in improving the optical transmittance and reliability of the LC devices. The TiO_2_ nanoparticles of 15 and 300 nm in size were surface-treated with stearic acid, and the PI mixture with TiO_2_ NPs was spin-coated on an ITO substrate and then imidized to form a hybrid alignment layer. The interactions between the long hydrophobic alkyl chains of stearic acid on the surface of the TiO_2_ NPs and the LC molecules was expected to improve the vertical alignment ability of the LC molecules, thereby improving the electro-optical properties of the device. It was confirmed that the contact angles of the hybrid PI layers were higher than that of the pure PI layer and the contact angle increased as the TiO_2_ content increased, indicating that the stearic acids treated on the surface of TiO_2_ NPs induced the hydrophobic surface of the hybrid PI layer. Similar trends were observed, irrespective of the size of the TiO_2_ NPs (15 or 300 nm). In addition, the dark state observed under cross-polarization indicated a complete vertical alignment of the LC device with hybrid PI layers, and the relatively high threshold voltage and fast decay response time of the device suggested the improved vertical alignment ability of the hybrid PI layer. In addition, the optical transmittance of LC cell was enhanced by incorporating TiO_2_ NPs into PI layer, and the transmittance increased as the size of the TiO_2_ NPs increased. The VHR and RDC measurements revealed that the hybrid PI layers provided high VHR and low RDC values due to the ion trap effect of TiO_2_ compared to those of pure PI layer. Moreover, the long-term driving evaluation indicated that the functional hybrid PI layer with TiO_2_ NPs afforded the improved reliability of the device. From the perspective of the alignment material, the proposed method using hybridization of organic PI and surface-treated TiO_2_ NPs is expected to have a significant impact on the display field because it can afford simple preparation process, stable vertical orientation of LCs, high optical transmittance, and long-term reliability of the device.

## Data Availability

Data is contained within the article.
